# Eschar Formation Following Coma Bullae: A Case Report

**DOI:** 10.7759/cureus.58646

**Published:** 2024-04-20

**Authors:** Divya Minnaganti, Arjun R Gampala, Yash P Khanna, Emily Haury

**Affiliations:** 1 Internal Medicine, University of Missouri Kansas City School of Medicine, Kansas City, USA; 2 Internal Medicine and Pediatrics, University of Missouri Kansas City School of Medicine, Kansas City, USA

**Keywords:** wound care, blistering skin disease, substance use, eschar, coma bullae

## Abstract

Coma blisters, or coma bullae, are lesions often seen in the setting of impaired consciousness. Most commonly associated with drug-induced comas, coma bullae have been repeatedly linked to central nervous system (CNS) depressing agents, such as opiates. These lesions are believed to develop due to a complex multifactorial process involving external pressure on the skin, which leads to hypoxia and eventual death of eccrine sweat glands. In addition, the vasoactive and inflammatory properties of CNS depressing agents may play a role in this process. Come bullae usually develop on pressure points 48-72 hours after the onset of impaired consciousness and are self-limiting. We present the case of a 68-year-old male who was brought to the emergency department after being found unresponsive on the street. The urine drug screen was positive for cocaine and fentanyl. The initial examination showed several large, non-tender bullae on his scalp that continued to expand over two days. He subsequently developed similar lesions on his thighs, right shoulder, and knuckles. Dermatology was consulted and clinically diagnosed the patient with coma bullae, likely attributed to his altered consciousness and opiate use. Notably, more violaceous bullae were found on the bilateral lower extremities, with dermatology suspecting additional vasculitic features related to concurrent opiate and cocaine use. Skin biopsy and aspiration were deferred to avoid the risk of infection, and the patient was discharged per dermatology’s recommendations for no immediate intervention. He continued to follow with wound care for the next six months, with most of the bullae healing. However, eschars developed over the scalp and left lower extremity, requiring debridement by general surgery. This case report underscores a unique manifestation of coma bullae. Unlike typical presentations localized to pressure-dependent areas and appearing after two to three days of unconsciousness, our patient exhibited blisters in atypical sites with associated vasculitic features. Moreover, the development of eschars over time may be linked to ongoing vasoactive drug use, reperfusion injury, and social determinants of health. This case highlights the complex and multifactorial nature of coma bullae, emphasizing the challenges in wound care and management despite their expected self-resolution.

## Introduction

Coma blisters, also known as coma bullae, describe tense, non-tender, bullous, and coalescing lesions that appear 48-72 hours after the onset of a coma or altered state of consciousness [1]. They were first reported in 1806 on comatose soldiers who had suffered carbon monoxide poisoning [1]. These lesions can result from any etiology of coma, including diabetes and chronic kidney disease [2]. They are most often, however, associated with drug-induced comas related to central nervous system (CNS) depressing agents, such as barbiturates, opiates, alcohol, hypnotics, tricyclic antidepressants, and antipsychotics [2]. Although there are several theories as to why coma bullae form, there is not a clear answer.

Coma bullae form most commonly on the pressure points of the skin [3]. For example, they tend to appear on the elbows, knees, ankles, and digits. Their diagnosis can be made clinically, but a skin biopsy showing subepidermal blisters with eccrine gland necrosis is definitive [1,3]. It is important to distinguish coma bullae from other similar bullous lesions such as friction or edema blisters, epidermolysis bullosa, and drug-induced or autoimmune bullous pemphigoid before deciding the course of action, as these diagnoses can present very similarly.

There is no specific treatment for coma bullae, and they typically resolve on their own within one to two weeks [2,3]. Management is typically supportive with appropriate wound care, decreasing the risk of infections and reversing the precipitating factor (e.g., drug overdose and diabetic ketoacidosis) [1,2]. Occasionally, topical antibiotics are used to prevent infections [3]. Rarely, bullae leave scars, such as those seen in our patient with the formation of an eschar [1].

## Case presentation

The patient was a 68-year-old male with a past medical history of chronic kidney disease, tobacco use, cocaine use, frostbite wounds, and housing insecurity who was brought to the emergency department for altered mentation. Vitals on arrival were significant for temperature of 101.2 degrees Fahrenheit, tachycardia of 110 beats per minute, and blood pressure of 170/102 mmHg. He had a Glasgow Coma Scale score of nine (out of 15). Labs showed a creatinine of 2.58 milligrams per deciliter (mg/dL), creatine kinase of 975 units per liter (U/L), and troponin of 843 nanograms per milliliter (ng/mL); the troponin then downtrended to 437 ng/mL. The urine drug screen was positive for cocaine and fentanyl and urinalysis was positive for blood. The patient's mental status improved after naloxone was administered.

**Figure 1:**
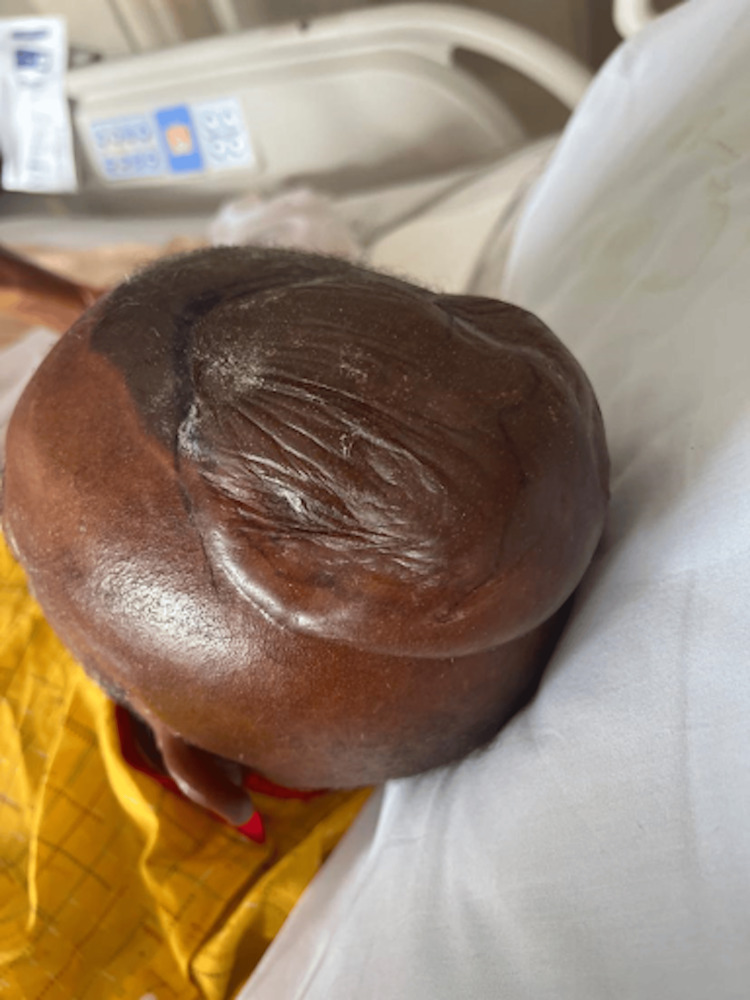
Bullae on the scalp at the time of initial presentation

**Figure 2:**
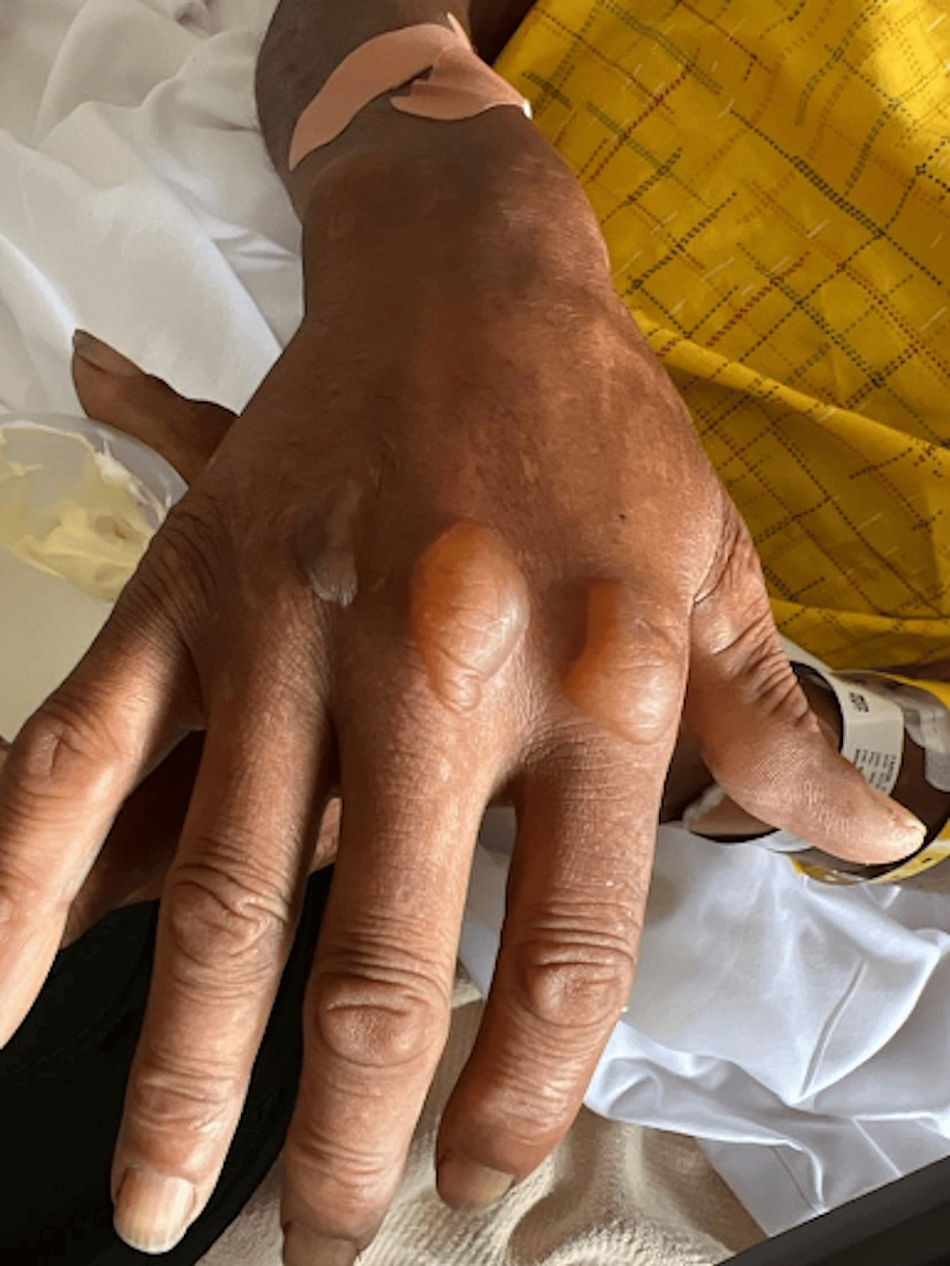
Bullae on the right hand at the time of the initial presentation

**Figure 3:**
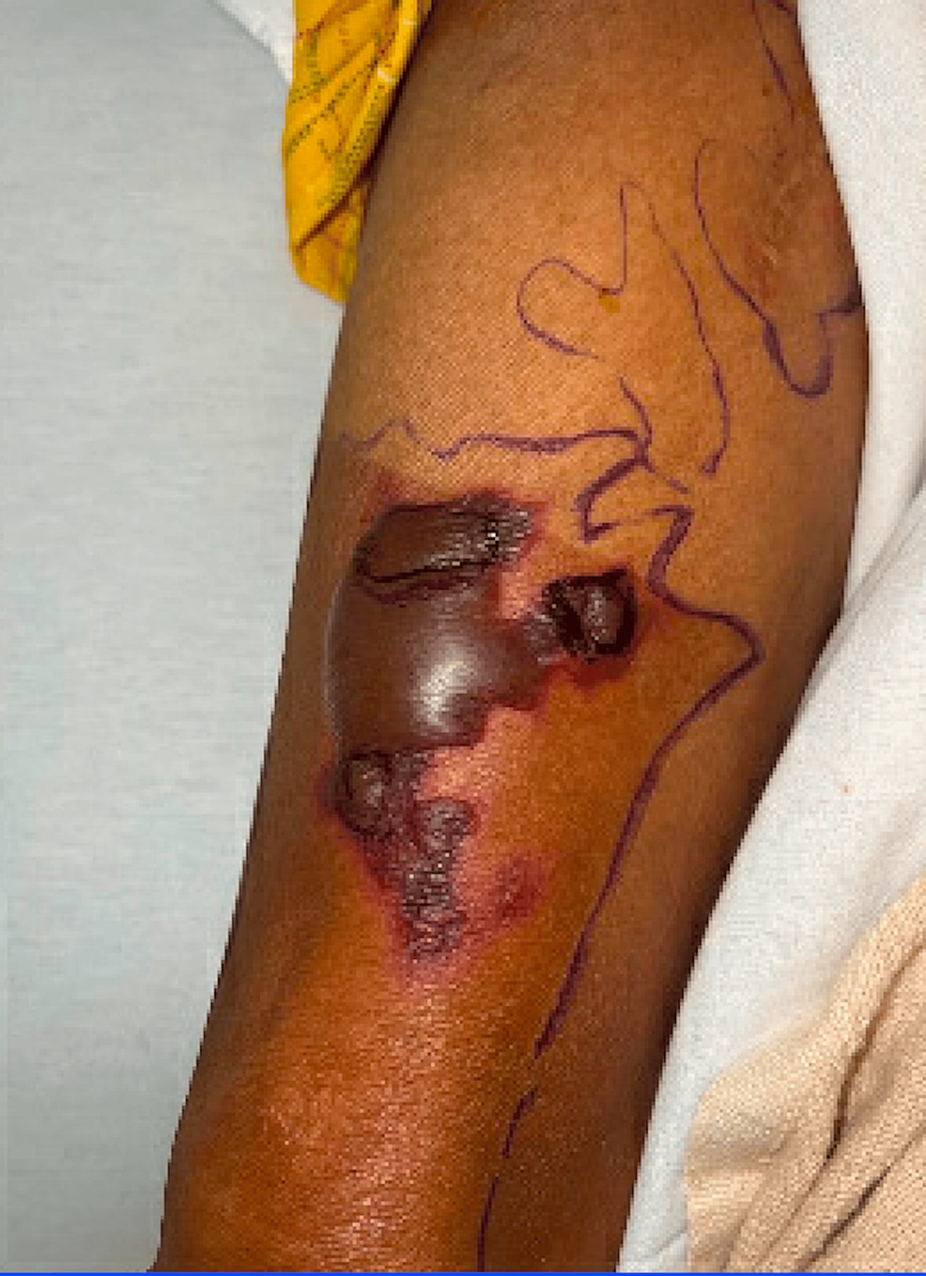
Bullae on the lower extremity with violaceous hue (per dermatology, this lesion is also vasculitic in nature)

**Figure 4:**
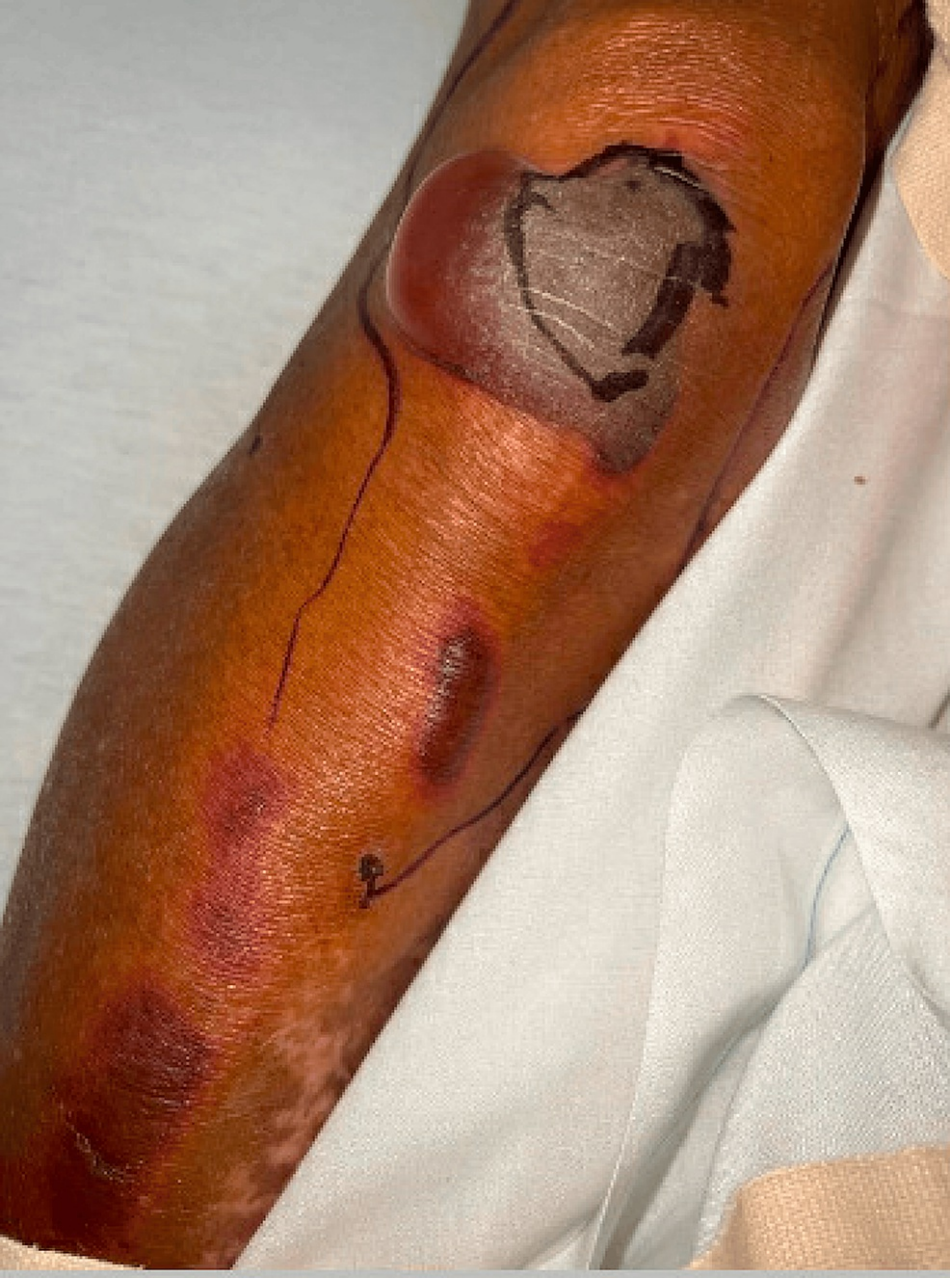
Bullae on the lower extremity with violaceous hue (per dermatology, this lesion is also vasculitic in nature)

On physical exam, the patient was noted to have numerous tense, non-inflammatory bullae with surrounding erythema covering the scalp (Figure 1), right hand (Figure 2), and abdomen. Over the course of his hospital admission, he developed more bullae on his right shoulder, left thigh, bilateral calves, and right metacarpals two, three, and four. The patient also had patchy erythema on his lower extremities that developed into tense, large bullae. The bullae appeared violaceous in color and were tender to palpation (Figures 3-4). The bullae on the top of the scalp eventually ruptured and drained serous fluid.

Blood cultures, serous fluid cultures, and antineutrophil cytoplasmic antibody (ANCA) were all ordered and came back negative. The patient denied any history of blistering skin conditions or reactions to medications causing skin-related manifestations. A dermatology consult was then placed, and a diagnosis of coma bullae was made based on the correlation of the patient’s fentanyl-positive urine drug screen, prior history of drug-induced altered mentation, and the morphology of skin lesions. Regarding the violaceous bullae observed on his bilateral lower extremities, dermatology indicated that these were likely coma bullae with associated vasculitic features, possibly attributable to the patient's concurrent fentanyl and cocaine use. Recommendations were made to continue supportive care and allow the lesions to resolve independently in two to four weeks. If the bullae were to open and spill their contents, the patient was told to place an absorbent dressing.

**Figure 5:**
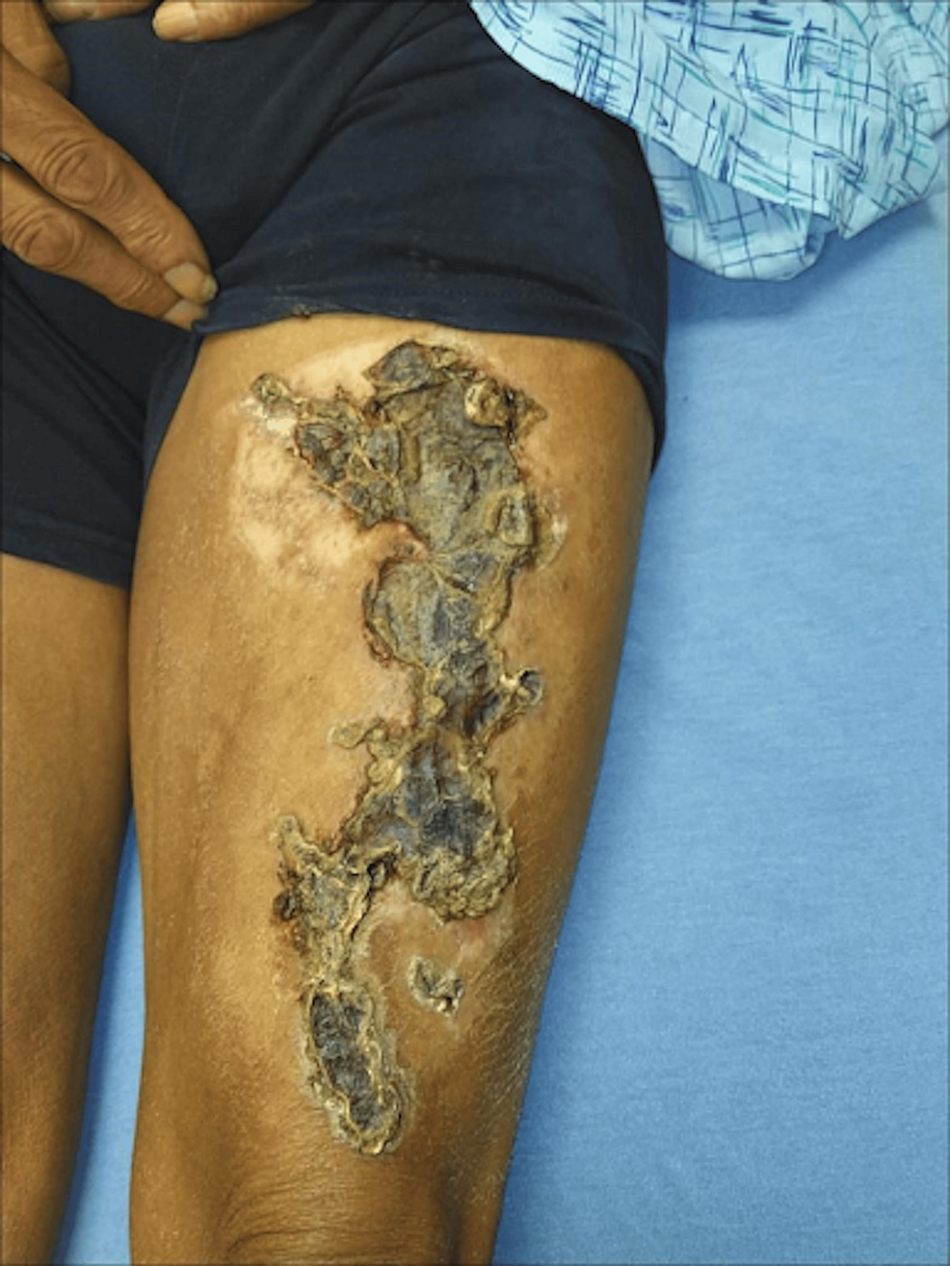
Bullae on the left thigh after eschar formation at a wound clinic appointment

**Figure 6:**
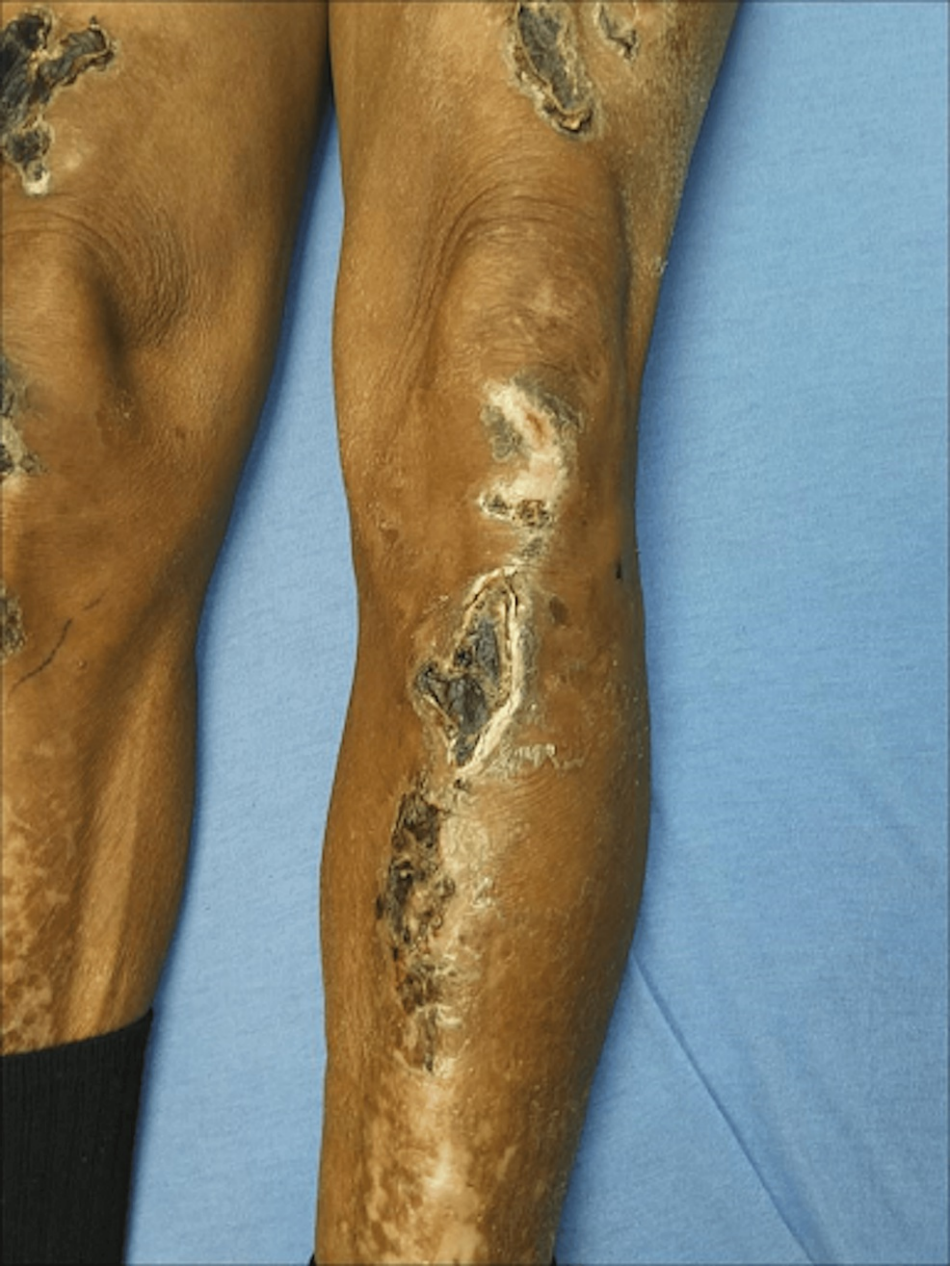
Bullae on the left calf after eschar formation at a wound care appointment

**Figure 7:**
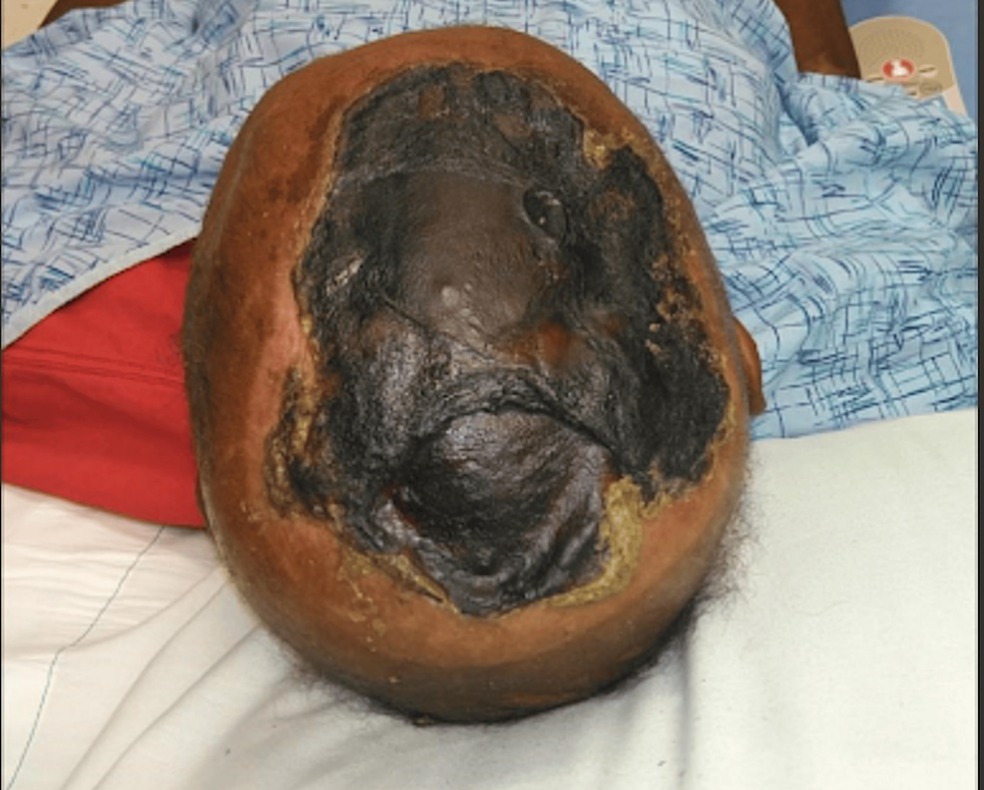
Bullae on the scalp after eschar formation at a wound clinic appointment

The patient was scheduled for a wound care evaluation two months after being discharged from the hospital. His skin lesions had formed into dry, hard brown eschars with an underlying malodorous yellow-colored discharge (Figures 5-7). He was told to perform frequent dressing changes, apply collagenase ointment to open/yellow areas, and cleanse all sites within and around the wounds with a pure hypochlorous acid solution. Due to the persistence of his lesions despite several weeks of therapy, general surgery performed an excisional debridement.

## Discussion

The etiology of coma bullae is unclear; however, several theories have been discussed in the literature. One of the central theories attributed to coma bullae revolves around prolonged external pressure on the skin, leading to vessel wall injury, decreased blood flow, and eventual hypoxia of the tissue [2]. With continual hypoxia to the area during a coma, necrosis of eccrine sweat glands, which lie in the dermis and epidermis, results in bullae formation. Another theory discusses the toxic effect of CNS depressing agents on the eccrine sweat glands, particularly with barbiturates and opiates. Some of these agents are thought to release inflammatory markers and activate the complement system, leading to inflammation and damage of eccrine sweat glands [2]. Many CNS depressing agents are also vasoactive, producing vasodilation; this reduces blood flow and results in tissue hypoxia [2]. In our patient's case, several bullae were noted on the anterior aspect of his extremities, which are typically considered pressure-dependent areas. While we lack information on whether the patient was initially found lying in a supine or prone position, the presence of bullae on the extremities aligns with the hypothesis of excessive pressure. However, the occurrence of a large bullous lesion on his scalp, a non-pressure-dependent area, suggests a different mechanism potentially related to the vasoactive and inflammatory properties of the drugs involved. Thus, coma bullae formation is likely multifactorial, involving an interaction of drugs, tissue ischemia, and trauma.

Our case represents an atypical case of coma bullae. The patient, who had a history of prolonged substance abuse, presented to the hospital with altered mental status. He was not oriented to location, suggesting he may have fallen unconscious. As discussed above, the patient had coma blisters in both pressure-dependent and independent areas, representing the multifactorial nature of these lesions. Moreover, the lesions on the lower extremities were identified as concurrently vasculitic in nature, exhibiting a violaceous hue that may signify a cocaine-induced vasculopathy alongside the coma bullae. This underscores the complexity of clinically diagnosing skin-blistering lesions, particularly due to overlapping characteristics. Therefore, differentiating coma bullae from other similar bullous lesions like friction or edema blisters, epidermolysis bullosa, drug-induced bullous pemphigoid, or autoimmune bullous pemphigoid is crucial before determining the appropriate course of action. In Table 1, we delineate and compare the distinguishing features of such overlapping skin blistering lesions.

**Table 1:** A comparison of key features and differences among coma bullae and similar bullous lesions, highlighting important etiological, clinical, histopathological, and diagnostic characteristics to aid in accurate differentiation and management decisions.

Feature/difference	Coma bullae	Friction or edema blisters	Epidermolysis bullosa	Drug-induced bullous pemphigoid	Bullous pemphigoid
Etiology	Multifactorial: CNS depressing agents; external pressure leading to hypoxia and eccrine gland necrosis [4]	Friction or pressure on the skin; accumulation of fluid in the epidermis [5]	Genetic mutations affecting skin structural proteins (e.g., collagen) [6]	Drug reaction triggering autoimmune response [7]	Autoimmune reaction targeting basement membrane proteins [8]
History	Associated with CNS depression; often in hospitalized or bedridden patients [4]	Related to repetitive friction or pressure; common in athletes or manual laborers [5]	Present from birth or early childhood; family history may be present [6]	Develops after drug exposure; may resolve upon discontinuation [7]	Usually seen in elderly; chronic, progressive course [8]
Clinical appearance	Large, tense bullae; often over bony prominences; eccrine gland involvement [4]	Small, superficial blisters; often localized to areas of repeated trauma [5]	Blisters and skin erosions; variable severity; may involve mucous membranes [6]	Large, tense bullae; may be widespread; associated with drug use history [7]	Large, tense bullae; typically on flexural surfaces; itchy, chronic lesions [8]
Histopathology	Necrosis of eccrine sweat glands; inflammatory infiltrate in the dermis [4]	Epidermal separation at the level of the stratum corneum or within the epidermis [5]	Subepidermal blisters; abnormal basement membrane or dermal-epidermal junction [6]	Subepidermal blistering; eosinophils and neutrophils in the dermis [7]	Subepidermal blistering; linear IgG and C3 deposits at the basement membrane zone [8]
Diagnosis	Clinical history and examination; biopsy for histopathological confirmation [4]	Clinical examination and history of repetitive trauma; no specific tests [5]	Genetic testing; skin biopsy for histopathology and immunofluorescence [6]	Drug history; biopsy findings consistent with pemphigoid [7]	Clinical presentation; biopsy with immunofluorescence studies [8]

While the clinical picture alone is often sufficient to diagnose coma bullae in the absence of any underlying blistering disorders or medication allergies, a skin biopsy may help to provide a definitive diagnosis. Under histologic evaluation, coma bullae have demonstrated findings of necrotic keratinocytes, thin epidermis, and degeneration of eccrine sweat glands [9]. In one study, immunohistochemistry analysis demonstrated positivity for CDK45, CK-L, and M30, which were markers found useful in evaluating sweat gland degeneration [10]. Direct immunofluorescence has been used to study coma bullae but usually has yielded negative results, as it is not clear if this pathology is immune-mediated [10]. Sometimes, the presence of inflammation in the blisters can be used to distinguish drug-induced from non-drug-induced coma bullae.

Coma bullae are commonly self-limiting, and treatment is typically not required. Sometimes, relieving pressure from the coma bullae lesions, sterile dressings to collect drainage, and topical antibiotics to prevent secondary bacterial infections are used in management [11]. As opposed to several other case reports in which the patients improved within a few weeks of diagnosis, the patient in this case proceeded to form eschars over the regions of his bullae along with purulent fluid. This complication recalls a case report of coma bullae associated with the brachioradial artery, where the patient’s bullae were also absorbed, leaving ulcers, necrosis, and violet patches. However, her lesions were healed with tissue debridement over two weeks [12]. While our patient did have appropriate follow-up and treatment with wound care following his hospital stay, he reported that he continued to use illicit substances. In addition, his living situation was unstable, and he was presumed to be living in unsanitary conditions. In the case of the coma bullae associated with the brachioradial artery, it was presumed that reperfusion injury after revascularization may have worsened the patient’s condition [12]. It could be theorized that the same reperfusion injury may have resulted in eschar formation in our patient, with his continued use of vasoactive drugs further depriving blood flow to the already hypoxic lesions that were healing. Many studies have shown that poor social determinants of health, such as the presumed unsanitary conditions in this patient's case, are likely contributory factors to his poor disease progression, although it is important to note that the study referenced did not specifically investigate the role of social determinants of health in coma bullae healing, but rather focused on healing outcomes more broadly [13].

## Conclusions

We report a unique case of drug-induced coma bullae in a patient with a history of extensive opiate drug use and altered consciousness upon hospital admission. Typically, coma bullae manifest after two to three days of unconsciousness and localize to pressure-dependent areas, but our patient presented with blisters in atypical sites; there were additional vasculitic features to some of the bullae. This observation suggests a novel manifestation of coma blisters, likely attributable to a combination of cocaine, opiates, and pressure injury during his unconscious state. Furthermore, coma bullae are usually self-resolving; the presence of eschar formation could be linked to ongoing vasoactive drug use, reperfusion injury, and social determinants of health.
